# Production of hemo- and immunoregulatory cytokines by erythroblast antigen^+ ^and glycophorin A^+ ^cells from human bone marrow

**DOI:** 10.1186/1471-2121-5-39

**Published:** 2004-10-18

**Authors:** Sergey V Sennikov, Tatyana V Injelevskaya, Sergey V Krysov, Alexandr N Silkov, Igor B Kovinev, Natalya J Dyachkova, Anton N Zenkov, Mary I Loseva, Vladimir A Kozlov

**Affiliations:** 1 Laboratory of the Regulation of Immunopoiesis, Institute of Clinical Immunology SB RAMS, Yadrintsevskaya 14, Novosibirsk, 630099, Russia; 2 The Department of Haematology, Regional Haematological Center, Novosibirsk, Russia

## Abstract

**Background:**

Erythroid nuclear cells (ENC) of the bone marrow (BM) have not previously been considered as important producers of wide spectrum of haemo- and immunoregulatory cytokines. The aim of the current work was to confirm the production of the main hemo- and immunoregulatory cytokines in human ENC from BM.

**Results:**

We used native human BM ENC in our experiments. We for the first time have shown, that the unstimulated erythroblasts (Gl A^+ ^or AG-EB^+^) produced a wide spectrum of immunoregulatory cytokines. Human BM ENC produce cytokines such as interleukn (IL)-1β, IL-2, IL-4, IL-6, interferon (IFN)-γ, transforming growth factor (TGF)-β1, tumor necrosis factor (TNF)-α and IL-10. They can be sub-divided into glycophorin A positive (Gl A^+^) and erythroblast antigen positive (AG-EB^+^) cells. To study potential differences in cytokine expression between these subsets, ENC were isolated and purified using specific antibodies to Gl A and AG-EB and the separated cells were cultivated for 24 hours. The cytokine contents of the supernatant were measured by electrochemiluminescence immunoassay. Quantitative differences in TGF-β1 and TNF-α production were found between Gl A^+ ^and AG-EB^+ ^BM ENC. Furthermore, in vitro addition of erythropoietin (EPO) reduced IFN-γ and IL-2 production specifically by the AG-EB^+ ^ENC. Thus, Gl A^+ ^and AG-EB^+ ^ENC produce IL-1β, IL-2, IL-4, IL-6, IFN-γ, TGF-β1 and TNF-α. Gl A^+ ^ENC also produce IL-10.

**Conclusion:**

Cytokine production by erythroid nuclear cells suggests that these cells might be involved in regulating the proliferation and differentiation of hematopoietic and immunocompetent cells in human BM.

## Background

Haematopoesis is regulated by lymphoid and non-lymphoid cells through a complex network of paracrine and autocrine mechanisms involving cytokines, growth factors and their receptors. However, although stromal [1-6], endothelial [7-10], megakaryocytic [11,12] and osteogenic cells [13-16] and lymphocytes are known to express cytokines, erythroid nuclear cells (ENC), the major cell population of the BM, are not considered as important producers of hemo- and immunoregulatory cytokines.

Experiments on ENC isolated from mouse spleen undergoing erythroid hyperplasia, and on cells separated from single erythroid colonies, have revealed that mRNAs for cytokines such as IL-1α, IL-1β, IL-4, IL-6, granulocyte-macrophage colony stimulating factor (GM-CSF), TGF-β1 and IFN-γ are present [17,18]. Production of GM-CSF and IFN-γ has also been reported [17]. Similarly, IL-2 and IL-3 mRNAs were found after erythroid cells were treated with erythropoietin (EPO) [17-20]. Human fetal liver erythroid cells have also been shown to produce such cytokines as IL-1, IL-2, IL-4, IL-6, IL-10, TNF-α, IFN-γ and TGF-β1 [20]. Stopka and co-authors have demonstrated the ability of burst-forming unit erythroid cells (BFU-E) to express and produce EPO [21]. Also it has been shown that erythroid cells express and produce vascular-endothelial growth factor (VEGF)-A and placental growth factor (PlGF) both in vitro and in vivo. Production of these proteins varies during differentiation and is increased 100-fold by PlGF and 3-fold by VEGF-A [22]. Macrophage colony stimulating factor (M-CSF), fibroblast growth factor (FGF)-2, VEGF-A, hepatocyte (H)GF, insulin-like (I)GF-1, IL-1β, trombopoietin (TPO), TNF-α, IFN-γ, FAS-L, and macrophage inflammatory protein (MIP)-1α mRNAs are also expressed in BFU-E isolated from BM, and VEGF-A and TGF-β1 are produced [23,24]. All these data allow us to consider erythroid cells as cytokine producers involved in regulating hemo- and immunopoiesis.

The aim of the current work was to confirm previous observations by studying the production of the main hemo- and immunoregulatory cytokines, IL-1β, IL-2, IL-4, IL-6, IL-10, TNF-α, IFN-γ and TGF-β1, in human ENC from BM. Furthermore, we wished to determine whether different subsets of ENC (AG-EB+ and Gl A+ erythroid cells from human BM) might account for altered expression levels. Expression of both these markers is specific to erythroid cells; they are absent from the membranes of other blood-forming cells. AG-EB is expressed in a proportion of BFU-E cells (up to 4% of AG-EB^+ ^BFU-E cells) and on colony-forming units – erythroid (CFU-E) (up to 36% of AG-EB^+ ^CFU-E cells) [25]. Its expression on erythroblasts is further increased (up to 90% in AG-EB^+^erythroblasts). Later in development, AG-EB expression decreases, and only 4% of reticulocytes carry AG-EB [25]. In contrast, Gl A is expressed in a minor subpopulation of the CFU-E cells (less than 4%). On the erythroblasts this expression is increased and reach up to 100% in reticulocytes [26,27]. Sheme of AG-EB and Gl A expression during erythroid maturation present in Table 1.

**Table 1:** Expression of AG-EB and Gl A during erythroid maturation. The percentage of positive cells detected by flow cytometric analysis [25, 26, 27].

	% of positive cells			
	
	BFU-E	CFU-E	Proerythroblasts → erythroblasts	reticulocytes
AG-EB	0 – 4	36	93–95	4
Gl A	0	0 – 4	Increase with maturation	100

In this article we show which human BM ENC produce the cytokines IL-1β, IL-2, IL-4, IL-6, IFN-γ, TGF-β1, TNF-α and IL-10.

## Results

**Figure 1:**
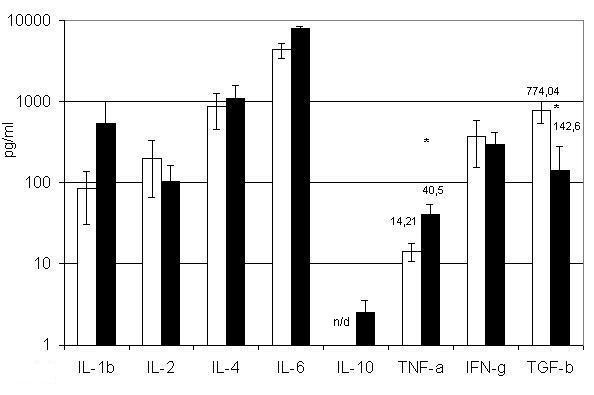
**Cytokine production by BM erythroid nuclear cells carrying Erythroblast Antigen and Glycophorin A. **□ – BM AG-EB^+ ^erythroid nuclear cells; ■ – BM Gl A^+ ^erythroid nuclear cells. All populations were cultivated for 24 h at concentrations of 10^6 ^cells per ml. Results present as (Mean ± SEM). n/d – not detected. * – P < 0.05.

Erythroid cells were separated according to the presence of the surface markers AG-EB and Gl A. BM erythroid cells carrying AG-EB produce the cytokines IL-1β, IL-2, IL-4, TNF-α, IFN-γ and TGF-β1 (figure 1). Those carrying Gl A also produce these cytokines and in addition might be secrete IL-10 (figure 1). Interestingly, erythroid cells carrying AG-EB release more TGF-β1 (p < 0.05) than Gl A^+ ^cells, whereas Gl A^+ ^secrete more TNF-α (p < 0.05).

To the best of our knowledge, this difference between BM AG-EB^+ ^and Gl A^+ ^ENC has not previously been reported. To verify that the cytokines were truly expressed by these erythroid cells, we isolated erythoid colonies by cultivating single BM cells in MethoCult H4433 medium. As shown in Table 2, the cytokines produced were comparable between ENC derived from erythroid colonies and ENC enriched from the BM.

**Table 2:** Concentration of cytokines in conditioned medium derived from erythroid cells. Concentration of cytokines (pg/ml) in conditioned medium derived from enriched population of BM erythroid cells (1 × 10^6^/ml) and erythroid cells isolated from erythroid colonies (1 × 10^6^/ml). Results were obtained from at least three independent experiments (Mean ± SEM).

	Enriched erythroid nuclear cell population	Erythroid nuclear cells derived from erythroid colonies
IL-1β	78.64 ± 44.84	10.8 ± 0.39
IL-2	255.36 ± 88	237.73 ± 237.73
IL-4	62.66 ± 45.28	738.26 ± 640.15
IL-6	741.08 ± 395.95	324 ± 56.14
IL-10	121.26 ± 53.22	73.4 ± 73.4
TNF-α	2.59 ± 1.41	0.0
IFN-γ	766.35 ± 319.45	752.88 ± 492.65
TGF-β1	524.88 ± 372.59	479.25 ± 267.75

**Figure 2:**
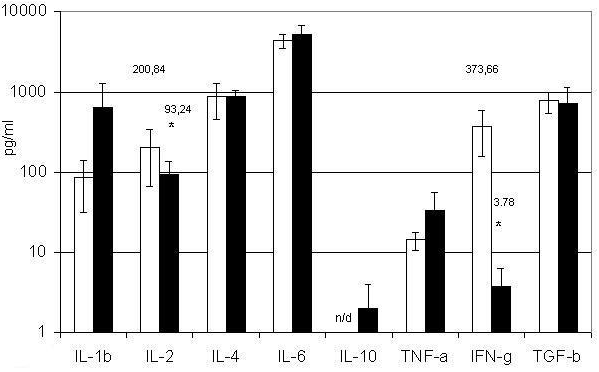
**Influence of EPO on cytokine production by BM AG-EB^+^erythroid nuclear cells. **□ – BM AG-EB^+ ^erythroid nuclear cells; ■ – BM AG-EB^+ ^erythroid nuclear cells after treatment with EPO. BM AG-EB^+ ^erythroid nuclear cells (10^6 ^cells/ml) were treated with EPO (2 U/ml) for 24 h. Results present as (Mean ± SEM). n/d – not detected. * – P < 0.05.

EPO regulates the proliferation and differentiation of erythroid cells [28-30]. Therefore, we assessed the effect of EPO on our different ENC cell populations and found that the production of some hemo- and immunoregulatory molecules was altered (figure 2). For instance, EPO treatment of AG-EB^+ ^cells led to a 99% decrease in IFN-γ production and a 50% decrease in IL-2 production, and the production of IL-1β and TNF-α was enhanced. However, EPO treatment had no discernible effect on the cytokine profile of Gl A^+ ^cells.

## Discussion

We have shown that the native BM ENC can produce a wide spectrum of cytokines, which are capable of both stimulating and inhibiting erythropoiesis. Cytokine production has been confirmed using ENC isolated from erythroid colonies cloned from a single BM cell.

Earlier we demonstrated cytokine production by ENC derived from fetal tissues [19,20]. However, there was a great difference between the profiles of cytokines released by BM and fetal ENC.

TGF-β1 is known to inhibit the proliferation of erythroid precursors and to provoke or accelerate the terminal differentiation of mature erythroid cells [31-33]. The fetal AG-EB^+ ^and Gl A^+ ^erythroid cells produced TGF-β1 in concentrations less than 5 pg/ml and we suggested that this could be related to the increased proliferative activity of erythroid cells in the fetal liver [20]. We propose that the high level of TGF-β1 production by BM erythroid cells is probably related to the need to restrain erythroid cell proliferation in healthy adult individuals. This hypothesis were declared also in review [24].

It is notable that BM erythroid cells produce not only inhibitors but also high levels of stimulators of erythropoiesis such as IL-4 and IL-6, especially in the presence of EPO [34,35]. The production of these hemoregulatory molecules suggests that erythroid cells have a capacity for self-regulation. Interestingly, IL-2, which inhibits erythropoiesis [36], is produced by these cells in smaller quantities or, like IL-10, is not produced at all.

Besides the differences in production of some cytokines by BM erythroid cells, there are parallels with cytokine production from fetal erythroid cells. A similar situation can be observed when TNF-α production by BM erythroid cells is compared with those from fetal liver [20]. In both cases AG-EB^+ ^cells produce significantly less TNF-α than Gl A^+ ^(figure 1). TNF-α is a known inhibitor of precursor cell proliferation and an inducer of cell death in a proportion of these cells [37,38]. Therefore it is plausible that erythroid cells use this cytokine for autocrine self-regulation, by maintaining a negative feedback loop. If this is so, then relatively small increases in the number of more differentiated Gl A^+ ^cells would lead to a decreased precursor proliferation rate, due to the elevated level of TNF-α. Negative feedback can also be maintained through IFN-γ, which is produced at the same level by both Gl A^+ ^and AG-EB^+ ^cells. However, IFN-γ initiates apoptosis only in BFU-E [39,40], while in CFU-E cells it increases the expression of Bcl-x and protects these cells from elimination. Thus, IFN-γ enhances the differentiation of erythroid precursors [41,42]. We found no statistical significant differences between AG-EB^+ ^and Gl A^+ ^cells in the production of IL-1β, IL-2, IL-4, IL-6 and IL-10.

The capacity of erythroid cells to change their cytokine expression profiles quantitatively and qualitatively under different conditions is an important indicator of their active role in the regulation of hemo- and immunopoiesis. Similar data were previously obtained in mouse cells and are presented here for human BM erythroid cells. Treatment with EPO leads to significant changes in IL-2 and IFN-γ production. Notably, IFN-γ and IL-2 secretion by AG-EB^+ ^cells is lowered in the presence of EPO, while Gl A^+ ^cells show no such change. This difference could be related to EPO-R expression in AG-EB^+ ^cells, which are mainly CFU-E and erythroblasts [43-45]. Expression of this receptor ceases in more differentiated early erythroblasts. Indeed, Gl A^+ ^cells do not express EPO-R, since late erythroblasts and more differentiated forms of erythroid cells are prevalent in this population [28,43,46]. The EPO-induced changes in cytokine production mainly concern inhibitors of erythroid precursor proliferation, such as IL-2 and IFN-γ. Thus, one mechanism by which erythroid cell proliferation is stimulated could be a decrease in proliferation inhibitor production by these cells as a result of EPO treatment.

## Conclusion

Erythroid nuclear cells appear to be active producers of hemo- and immunoregulatory cytokines, involved in regulating the proliferation and differentiation of hematopoietic and immunocompetent cells in human BM. Changes in the cytokines produced by erythroid cells in response to EPO suggests the ability of these cells to respond to microenvironmental changes by altering the cytokine production profile.

## Methods

### BM cells

This work was approved by the local ethics committee. After obtaining informed consent, sternum BM samples were collected from 8 normal healthy volunteers. All samples used in this study exhibited normal myelograms. The enriched population of erythroid cells was isolated from BM mononuclear cells by depleting of adherent cells and granulocytes as described previously [19].

### Isolation of erythroid cell population carrying a surface erythroid antigen and Glycophorin A

We used indirect panning to obtain cells expressing either the erythroid antigen (AG-EB) or Glycophorin A (Gl A) [19]. Monoclonal mouse anti-red blood cell Glycophorin A (MAS518, Harlan-Sera Lab, England), and monoclonal HAE-9 antibodies against AG-EB (kindly provided by Prof. Mechetner, Russia) were used [25]. Affinity isolated polyclonal rabbit anti-mouse immunoglobulin (Biosan, Russia) was used as a secondary reagent to coat Petri dishes during the panning procedure. To avoid non-specific binding to FcRs, cells were blocked with aggregated normal human IgG and then incubated with anti-Gl A or HAE-9 antibodies for 50 min at 4°C. Cells were transferred to Petri dishes covered with rabbit anti-mouse antibodies for panning for 50 min at 4°C. Subsequently, immobilised cells were collected, cultivated for 24 h in RPMI-1640 with 10% horse serum (Sigma, USA) with and without EPO [19,47]. Samples were collected after cultivation.

### Cultivation of erythroid cells with EPO

1 × 10^6 ^cells were placed in culture medium containing 2 U/ml Epo (Boehringer Mannheim, Germany) for a 24-h incubation.

### CFU-E

Cells from the enriched erythroid population were maintained as described above [19,20] at 2·10^5^/ml and then cultivated in MethoCult H4433 medium (Stemcell Tech. Inc. Vancouver, B.C.) for 14 days. Colonies were visually characterised as CFU-E using an inverted microscope. After cultivation, the colonies were harvested and washed twice to remove methyl cellulose. The resulting cellular suspension (1 × 10^6^/ml) was cultivated for 24 h in RPMI-1640 with 10% horse serum, then supernatants were collected. Samples were stored at -20°C.

### Cells purity

Cell purity was assessed using Nocht-Maksimov staining of smears [48] and staining of haemoglobin with benzidin [49]. All cells (Gl A^+^, AG-EB^+ ^and CFU-E cells) were characterised as erythroblasts and were haemoglobin positive.

### Electrochemiluminescence (ECL) method for quantitative determination of cytokines

Quantitative determination of cytokines was performed by the electrochemiluminescent immunoassay [50-52] using an ORIGEN Analyzer (IGEN Inc., USA) according to the manufacturer's protocol. Calibration curves ranged from 10 to 10,000 pg/ml. Assay sensitivity was 2.8 pg/ml, 2 pg/ml, 2 pg/ml and 6 pg/ml for IL-1β, IL-2, IL-4 and IL-6, respectively. Assay sensitivities were 2 pg/ml, 1 pg/ml, 1 pg/ml and 2 pg/ml for IL-10, TNF-α, IFN-γ, and TGF-β1, respectively. The ruthenylated and biotinylated antibodies were diluted to working concentrations (μg/ml) 1:1, 1:1, 1:2 and 2:1 for IL-1β, IL-2, IL-4 and IL-6, respectively. The biotinylated and ruthenylated antibodies were diluted to working concentrations (μg/ml) 2:2, 1:1, 2:1 and 4:2 for IL-10, TNF-α, IFN-γ and TGF-β1, respectively.

For TGF-β1 detection, samples were treated with HCl to obtain intact and easily detectable proteins as described [53].

### Antibodies

All polyclonal and monoclonal antibodies were purchased from R&D Systems (Abington, UK). The following antibodies were used: against recombinant IL-1β, IL-2, IL-4, IL-6, IL-10, TNF-α, IFN-γ, TGF-β1 polyclonal and monoclonal: anti-hIL-1β #AB-201-NA, anti-hIL-1β #MAB201, anti-hIL-2 #AB-202-NA, anti-hIL-2 #MAB202, anti-hIL-4 #AB-204-NA, anti-hIL-4 #MAB204, anti-hIL-6 #AB-206-NA, anti-hIL-6 #MAB206, anti-hIL-10 #AB-217-NA, anti-hIL-10 #MAB217, anti-hTNF-α #AB-210-NA, anti-hTNF-α #MAB210, anti-hIFN-γ #AB-285-NA, anti-hIFN-γ #MAB285, anti-hTGF-β1 #AF-101-NA, anti-hTGF-β1 #MAB240. No antibody used displayed cross-reactivity with other cytokines.

### Cytokines

Recombinant cytokines were purchased from R&D Systems (Abington, UK): rhIL-1β #201-IL, rhIL-2 #202-IL, rhIL-4 #204-IL, rhIL-6 #206-IL, rhTNF-α #210-TA, rhIFN-γ #285-IF, rhTGF-β1 #240-B-002; and PeproTech, Inc. (Rocky Hill, NJ): rhIL-10 #200-10 was used for calibration curves. All the recombinant cytokines were diluted to 2 μg/ml in PBS, pH 7.4, supplemented with 0.05% NaN_3 _and 0.1% BSA (aliquots were kept at -70°C before use).

### Statistical analysis

Results are presented as means ± SEM. Statistical significance was determined using the nonparametric Mann-Whitney U-test. P < 0.05 was considered to be significant.

## Authors' contributions

IBK, NJD, ANZ and MIL carried out the selection of donors, sternum BM sampling and analysis of myelograms. TVI, ANS and SVK carried out all of the experiments and drafted the manuscript. SVS participated in design and coordination of the research and revision of the manuscript. ANS participated in the preparation and revision of the manuscript. VAK conceived of the study. All authors read and approved the final manuscript.
